# Association between blood lipid levels, BMI, and hypertension among employees in petrochemical enterprises

**DOI:** 10.3389/fcvm.2026.1788552

**Published:** 2026-04-01

**Authors:** Qingsong Li, Haobiao Liu, Xuefeng Yu, Jing Tang, Licheng Yang, Yingjie Cai, Zhihao Yang, Lianxu Jia, Ziwei Guo, Jing Han

**Affiliations:** 1 Xi'an Gem Flower Changqing Hospital, Xi'an, Shaanxi, China; 2 Department of Occupational and Environmental Health, School of Public Health, Health Science Center, Xi'an Jiaotong University, Xi'an, Shaanxi, China

**Keywords:** BMI, dyslipidemia, hypertension, non-traditional lipid markers, petrochemical enterprises

## Abstract

**Objective:**

To assess the combined effects of dyslipidemia, lipid parameters, non-traditional lipid markers, and body mass index (BMI) on hypertension incidence among petrochemical workers.

**Methods:**

This retrospective cohort study analyzed associations between lipid markers, BMI, and incident hypertension using multivariable logistic regression to estimate odds ratios (ORs) and 95% confidence intervals (CIs). Dose–response relationships were examined with restricted cubic spline models.

**Results:**

Dyslipidemia was associated with increased hypertension risk, with stronger associations in men. Elevated TC, TG, and LDL-C were significantly associated with hypertension in men, whereas only TG was significant in women. High LDL-C independently increased hypertension risk in the total population and in men. Increasing quartiles of TC and TG showed clear linear dose–response relationships. In men, hypertension risk rose with the number of coexisting lipid abnormalities. Non-traditional lipid markers (non-HDL-C, TG/HDL-C ratio, and TyG index) were positively associated with hypertension and exhibited linear dose–response patterns. Overweight and obesity further increased risk, particularly when combined with dyslipidemia.

**Conclusion:**

Adverse lipid profiles, non-traditional lipid markers, and excess body weight are important determinants of hypertension risk in this occupational cohort.

## 1 Introduction

Hypertension is a major global public health challenge and one of the most important risk factors for the development of cardiovascular disease, which remains the leading cause of morbidity and mortality worldwide (1–3). Elevated blood pressure contributes not only to coronary artery disease, stroke, and heart failure but also to kidney disease and other vascular complications (4, 5). The global burden of hypertension has increased steadily over the past decades, reflecting rapid urbanization, population aging, and lifestyle transitions. According to the *Global Report on Hypertension 2025*, a total of 140 million people worldwide were affected by hypertension in 2024, but only one in five people can control their condition through medication or lifestyle changes. In China, hypertension has emerged as a particularly pressing issue. National survey data indicate that the standardized prevalence of hypertension among Chinese adults aged 18–69 years first rose from 20.8% in 2004 to 29.6% in 2010, before declining to 24.7% in 2018 (6). Meanwhile, nearly half of Chinese adults aged 35–75 years in China have hypertension (44.7%) (7). This growing burden highlights the need to identify population-specific determinants of hypertension to develop more precise prevention and intervention strategies.

Occupational populations represent an important context for cardiovascular risk assessment, as work-related lifestyle patterns and health surveillance environments may influence metabolic risk profiles. Petrochemical workers, in particular, constitute a vulnerable group due to long-term exposure to multiple occupational hazards (8, 9). These include high ambient temperatures, excessive noise, toxic chemical agents, as well as adverse work practices such as shift work, prolonged sitting, and irregular schedules. Previous epidemiological investigations have suggested that such exposures may disrupt circadian rhythms, induce chronic stress, impair lipid metabolism, and increase oxidative stress, all of which can elevate the risk of metabolic disorders and hypertension (10, 11). Consistent with this, recent study has shown that petrochemical workers experience a significantly higher prevalence of metabolic disorders than the general population, making them an occupational group warranting special attention. Indeed, the prevalence of hyperlipidemia in petrochemical and gas workers has been reported to exceed 50%, while hypertension prevalence surpasses 30% (12). These statistics underscore the urgency of understanding how modifiable metabolic risk factors, such as lipid abnormalities and overweight/obesity, contribute to hypertension in this occupational population.

Although the link between dyslipidemia and hypertension has been widely acknowledged, most prior studies have concentrated on two specific areas: overall dyslipidemia diagnosis and elderly populations (13, 14). As a result, two key knowledge gaps remain. First, there is a paucity of research examining the role of individual lipid markers, including both traditional markers such as low-density lipoprotein cholesterol (LDL-C) and triglycerides (TG), and non-traditional markers such as non-high-density lipoprotein cholesterol (non-HDL-C) and the triglyceride-glucose (TyG) index—in relation to hypertension among petrochemical workers. Understanding these associations is particularly important because lipid markers represent not only diagnostic indicators but also potential intervention targets. Second, despite the well-established independent roles of dyslipidemia and excess adiposity in the development of hypertension, their combined effect remains poorly studied in high-risk occupational groups. Specifically, the extent to which dyslipidemia may exacerbate the hypertensive risk conferred by overweight or obesity, or vice versa, is not well understood. This lack of evidence hampers the formulation of precise occupational health guidelines and tailored interventions for petrochemical workers.

Therefore, the present study was designed to systematically analyze the associations between lipid markers, BMI, and hypertension in petrochemical workers. By investigating both traditional and non-traditional lipid indicators, as well as the combined impact of dyslipidemia and overweight/obesity, this study aims to fill critical gaps in the literature and to provide evidence-based insights into hypertension prevention and control in occupational populations. Clarifying these relationships is expected to contribute not only to occupational health management in the petrochemical industry but also to broader public health efforts targeting hypertension in other high-risk groups.

## 2 Materials & methods

### 2.1 Study population

This retrospective cohort study consisted of petrochemical workers who underwent occupational health examinations at a medical examination center in Xi'an from January 2014 to December 2015 (baseline). The subsequent period, which was defined as the follow-up period, spanned from January 2016 to December 2019, with December 31, 2019, designated as the follow-up endpoint. The inclusion criteria for this study are as follows: (1) No history of hypertension or antihypertensive medication use at the time of the baseline evaluation; (2) Complete data for height, weight, systolic blood pressure, diastolic blood pressure, fasting plasma glucose (FPG), TC, TG, HDL-C and LDL-C (individuals with missing data for any of these variables were excluded via listwise deletion/complete case analysis); (3) baseline age of less than 60 years (active employees); and a follow-up duration of at least two years. The following criteria were used to determine exclusion from the study: The development of a major mental illness during the follow-up period is the first consideration. The second consideration is the loss to follow-up due to non-participation or the absence of information regarding a hypertension diagnosis. Initially, 4,192 participants were enrolled in the study. Following the exclusion of 564 participants with baseline hypertension and 610 who were lost to follow-up, the final sample size was 3,018 (Supplementary Figure S1). This study was approved by the Ethics Committee of Xi'an Jiaotong University Health Science Center. As a retrospective study with anonymously collected data, informed consent from participants was waived.

### 2.2 Health indicators

Dyslipidemia: According to the 2023 China Guidelines for Lipid Management, dyslipidemia is defined as meeting any one of the following criteria: The following criteria must be met: TC ≥ 5.20 mmol/L, TG ≥ 1.70 mmol/L, HDL-C < 1.00 mmol/L, LDL-C ≥ 3.40 mmol/L, or a documented history of dyslipidemia accompanied by ongoing medication (15, 16). On traditional lipid markers: The non-HDL-C level is calculated as the difference between TC and HDL-C (17). The TG/HDL-C ratio is determined by dividing the TG level by the HDL-C level. The TyG index is calculated by the following formula: ln (TG × FPG)/2, where TG is in milligrams (mg) and FPG is in mg (18). Lipid Aggregation: The classification system is divided into four categories, contingent on the number of abnormal indicators observed among TC, TG, HDL-C, and LDL-C: 0 (all normal), 1 (1 abnormal), 2 (2 abnormal), and ≥3 (3 or more abnormal). The relationship between BMI and the concepts of overweight and obesity. The BMI is calculated using the following formula: BMI = weight (kilograms)/height² (meters squared). According to the Chinese Guidelines for Prevention and Control of Overweight and Obesity in Adults, BMI ≥ 24.00 kg/m² is defined as overweight/obesity (19). Hypertension: Diagnosis of hypertension is made if any one of the following criteria is met: The inclusion criteria for this study are as follows: Systolic blood pressure ≥140 mmHg, as measured in three consecutive measurements on the same day; Diastolic blood pressure ≥90 mmHg, as measured in three consecutive measurements on the same day; and Presence of antihypertensive medication, irrespective of blood pressure measurements (even if blood pressure <140/90 mmHg) (20, 21).

### 2.3 Statistical analysis

The database was established and subsequently cleaned before being imported into R 4.3.3 for statistical analyses. Because the exact timing of hypertension onset between the baseline and follow-up examinations could not be precisely determined, logistic regression was employed rather than survival analysis.

The following descriptive analysis was conducted: For quantitative data, normally distributed variables were expressed as mean ± standard deviation (SD). Intergroup comparisons employed independent samples t-tests. Non-normally distributed variables were expressed as median and interquartile range [M (P25, P75)], with intergroup comparisons utilizing the Wilcoxon signed-rank test. The count data were expressed as frequency (N) and proportion (%). The statistical analysis of intergroup differences was conducted by employing the chi-square test.

Association Analysis: Three logistic regression models were established with new-onset hypertension as the dependent variable and lipid parameters and BMI as independent variables. Model 1 (not adjusted for any variables), Model 2 (adjusted for gender, age, marital status, BMI, occupation, carotid atherosclerosis, FPG), Model 3 (Model 2 further adjusted for uric acid, alanine aminotransferase, alkaline phosphatase) The strength of the association was assessed by calculating odds ratio (OR) and 95% confidence interval (CI).

The lipid markers were then grouped into quartiles (Q1-Q4), with Q1 designated as the reference group. The linear relationship between lipid levels and hypertension risk was assessed via trend tests. RCS analysis was employed to evaluate the dose-response relationship between lipid levels and hypertension risk, with all available covariates taken into account in the analysis. All analyses employed two-sided tests at a significance level of *α* = 0.05.

## 3 Results

### 3.1 Baseline characteristics of study participants

Among the 3,018 participants included in the present study, the mean age was 38.02 ± 8.42 years, and 76.8% were male. A total of 1,714 individuals (56.8%) were classified as having dyslipidemia, while 1,304 (43.2%) exhibited normal lipid profiles. Compared with participants with normal lipid levels, those with dyslipidemia showed significantly higher proportions of males (79.60%), married individuals (86.35%), oil extraction workers (71.93%), diabetes (2.61%), and carotid atherosclerosis (14.80%) (all *P* < 0.05). Furthermore, baseline values of age, body mass index (BMI), systolic blood pressure, diastolic blood pressure, and fasting plasma glucose (FPG) were significantly greater in the dyslipidemia group than in the normal lipid group (*P* < 0.05) (Table 1).

**Table 1:** Comparison of baseline characteristics between groups with normal and abnormal blood lipids.

Characteristic	Blood lipid		*P*
	Normal	Abnormal	
Gender			<0.001
Female	983 (57.35)	266 (20.40)	
Male	731 (42.65)	1,038 (79.60)	
Marriage			<0.001
Married	1,353 (78.94)	1,126 (86.35)	
Unmarried/other	361 (21.06)	178 (13.65)	
Job type			<0.001
Oil production position	1,099 (64.12)	938 (71.93)	
Transportation/station control position	350 (20.42)	122 (9.36)	
Others	265 (15.46)	244 (18.71)	
Diabetes			<0.001
No	1,694 (98.83)	1,270 (97.39)	
Yes	20 (1.17)	34 (2.61)	
Carotid atherosclerosis			<0.001
No	1,576 (91.95)	1,111 (85.20)	
Yes	138 (8.05)	193 (14.80)	
Age (years)	35.10 ± 7.95	37.97 ± 8.49	<0.001
Age group (years)			<0.001
20–29			
30–39	721 (42.07)	516 (39.57)	
≥40	478 (27.89)	540 (41.41)	
BMI (kg/m^2^)	21.82 ± 2.90	24.74 ± 2.98	<0.001
BMI group (kg/m^2^)			<0.001
<24.00	1,349 (78.70)	532 (40.80)	
≥24.00	365 (21.30)	772 (59.20)	
SBP (mmHg)	111.06 ± 11.70	117.15 ± 11.20	<0.001
DBP (mmHg)	67.77 ± 8.60	72.12 ± 8.22	<0.001
FPG (mmol/L)	4.56 ± 0.49	4.72 ± 0.66	<0.001
ALP (U/L)	62.84 ± 17.20	73.28 ± 18.55	<0.001
GPT (U/L)	16.74 ± 11.20	25.45 ± 15.76	<0.001
GOT (U/L)	23.72 ± 6.76	27.70 ± 8.14	<0.001
GGT (U/L)	21.09 ± 17.22	37.64 ± 28.34	<0.001
Urea (mmol/L)	4.72 ± 1.30	5.06 ± 1.30	<0.001
Uric acid (umol/L)	294.21 ± 78.81	359.14 ± 86.00	<0.001
Creatinine (umol/L)	81.63 ± 11.27	87.88 ± 10.68	<0.001

Of the total participants, 1,137 (37.7%) were categorized as overweight or obese, and 1,881 (62.3%) had a normal BMI. Compared with individuals with normal BMI, those classified as overweight or obese had significantly higher proportions of males (77.57%), married individuals (87.07%), oil extraction workers (73.44%), diabetes (2.46%), and carotid atherosclerosis (14.86%) (all *P* < 0.05). Additionally, baseline age, systolic and diastolic blood pressure, and FPG levels were significantly elevated in the overweight/obesity group relative to the normal BMI group (*P* < 0.05) (Supplementary Table S1).

### 3.2 Prevalence of hypertension by lipid indicators

From the perspective of individual lipid indicators, participants with abnormal TC, TG, HDL-C, or LDL-C levels showed a significantly higher prevalence of hypertension compared with those with normal levels (26.20% vs. 15.42%, 24.57% vs. 12.83%, 20.45% vs. 15.32%, 26.01% vs. 14.97%, all *P* < 0.05). In women, abnormal TG was associated with a higher hypertension incidence (13.97% vs. 7.73%, *P* = 0.013). In men, abnormal TC, TG, and LDL-C levels were linked with higher hypertension prevalence (31.21%, 26.52%, and 29.85%, respectively) compared with those with normal values (20.74%, 18.35%, and 20.32%, all *P* < 0.05). No significant difference was observed for HDL-C levels (*P* = 0.414) (Supplementary Table S2).

### 3.3 Association between lipid profiles and hypertension

Multivariable logistic regression analysis was performed to assess the association between dyslipidemia and the risk of hypertension. In Model 1, dyslipidemia was significantly associated with increased hypertension risk in the total population (OR = 2.038, 95% CI = 1.675–2.480), in women (OR = 1.705, 95% CI = 1.098–2.646), and in men (OR = 1.442, 95% CI = 1.139–1.824; all *P* < 0.05). However, this association became nonsignificant in Models 2 and 3 (*P* > 0.05).

Regarding TC, high levels were significantly associated with elevated hypertension risk in the total population (OR = 1.948, 95% CI = 1.426–2.661) and among men (OR = 1.734, 95% CI = 1.230–2.445; *P* < 0.05). This association persisted in Model 2 but disappeared in Model 3. Similarly, elevated TG were significantly related to hypertension risk across all populations in Model 1 (OR = 2.213, 95% CI = 1.813–2.701; women: OR = 1.939, 95% CI = 1.139–3.303; men: OR = 1.606, 95% CI = 1.280–2.016; all *P* < 0.05), but not in Models 2 and 3 (*P* > 0.05).

Furthermore, low HDL-C was significantly associated with hypertension risk in the total population in Model 1 (OR = 1.420, 95% CI = 1.121–1.799; *P* < 0.05), but this association was not significant in subsequent models. Finally, elevated LDL-C levels showed a persistent positive association with hypertension in the total population (OR = 1.997, 95% CI = 1.536–2.597) and in men (OR = 1.669, 95% CI = 1.248–2.231; all *P* < 0.05), remaining significant across Models 2 and 3, whereas no association was found in women (*P* > 0.05) (Table 2).

**Table 2:** Relationship between dyslipidemia and hypertension risk in three different models.

Lipid profile	Model 1		Model 2		Model 3	
	OR (95%CI)	*P*	OR (95%CI)	*P*	OR (95%CI)	*P*
Total						
Blood lipids NL	—		—		—	
Blood lipids ABN	2.038 (1.675, 2.480)	<0.001^a^	1.073 (0.857, 1.343)	0.541	1.037 (0.825, 1.303)	0.755
TC NL	—		—		—	
TC ABN	1.948 (1.426, 2.661)	<0.001^a^	1.415 (1.020, 1.962)	0.038^a^	1.357 (0.976, 1.888)	0.07
TG NL	—		—		—	
TG ABN	2.213 (1.813, 2.701)	<0.001^a^	1.220 (0.976, 1.526)	0.081	1.167 (0.929, 1.467)	0.184
HDL-C NL	—		—		—	
HDL-C ABN	1.420 (1.121, 1.799)	0.004^a^	0.875 (0.679, 1.127)	0.301	0.885 (0.686, 1.141)	0.345
LDL-C NL	—		—		—	
LDL-C ABN	1.997 (1.536, 2.597)	<0.001^a^	1.467 (1.115, 1.931)	0.006^a^	1.426 (1.081, 1.883)	0.012^a^
Female						
Blood lipids NL	—		—		—	
Blood lipids ABN	1.705 (1.098, 2.646)	0.017^a^	1.080 (0.670, 1.739)	0.753	0.958 (0.583, 1.573)	0.864
TC NL	—		—		—	
TC ABN	1.326 (0.555, 3.170)	0.526	1.085 (0.442, 2.660)	0.859	0.974 (0.393, 2.415)	0.954
TG NL	—		—		—	
TG ABN	1.939 (1.139, 3.303)	0.015^a^	1.219 (0.689, 2.157)	0.497	1.086 (0.601, 1.962)	0.786
HDL-C NL	—		—		—	
HDL-C ABN	1.059 (0.518, 2.167)	0.874	0.757 (0.360, 1.592)	0.462	0.699 (0.328, 1.488)	0.353
LDL-C NL	—		—		—	
LDL-C ABN	1.666 (0.830, 3.341)	0.151	1.122 (0.543, 2.315)	0.756	1.037 (0.498, 2.157)	0.923
Male						
Blood lipids NL	—		—		—	
Blood lipids ABN	1.442 (1.139, 1.824)	0.002^a^	1.072 (0.833, 1.380)	0.59	1.047 (0.810, 1.354)	0.724
TC NL	—		—		—	
TC ABN	1.734 (1.230, 2.445)	0.002^a^	1.484 (1.042, 2.114)	0.029^a^	1.428 (0.999, 2.043)	0.051
TG NL	—		—		—	
TG ABN	1.606 (1.280, 2.016)	<0.001^a^	1.236 (0.970, 1.575)	0.086	1.187 (0.926, 1.521)	0.175
HDL-C NL	—		—		—	
HDL-C ABN	1.114 (0.860, 1.442)	0.414	0.901 (0.688, 1.180)	0.449	0.921 (0.702, 1.208)	0.552
LDL-C NL	—		—		—	
LDL-C ABN	1.669 (1.248, 2.231)	0.001^a^	1.528 (1.134, 2.061)	0.005^a^	1.496 (1.106, 2.025)	0.009^a^

NL, normal; ABN, abnormal. —: reference group. Model 1: No variables adjusted; Model 2: Adjusted for age, gender, marriage, BMI, job type, carotid atherosclerosis, and FPG; Model 3: Adjusted for age, gender, marriage, BMI, job type, carotid atherosclerosis, FPG, uric acid, alanine aminotransferase, and alkaline phosphatase. When stratified by gender, gender was not adjusted in Models 2 and 3.

a Statistically significant at 0.05 level.

### 3.4 Dose–response relationship between lipid levels and hypertension

**Figure 1:**
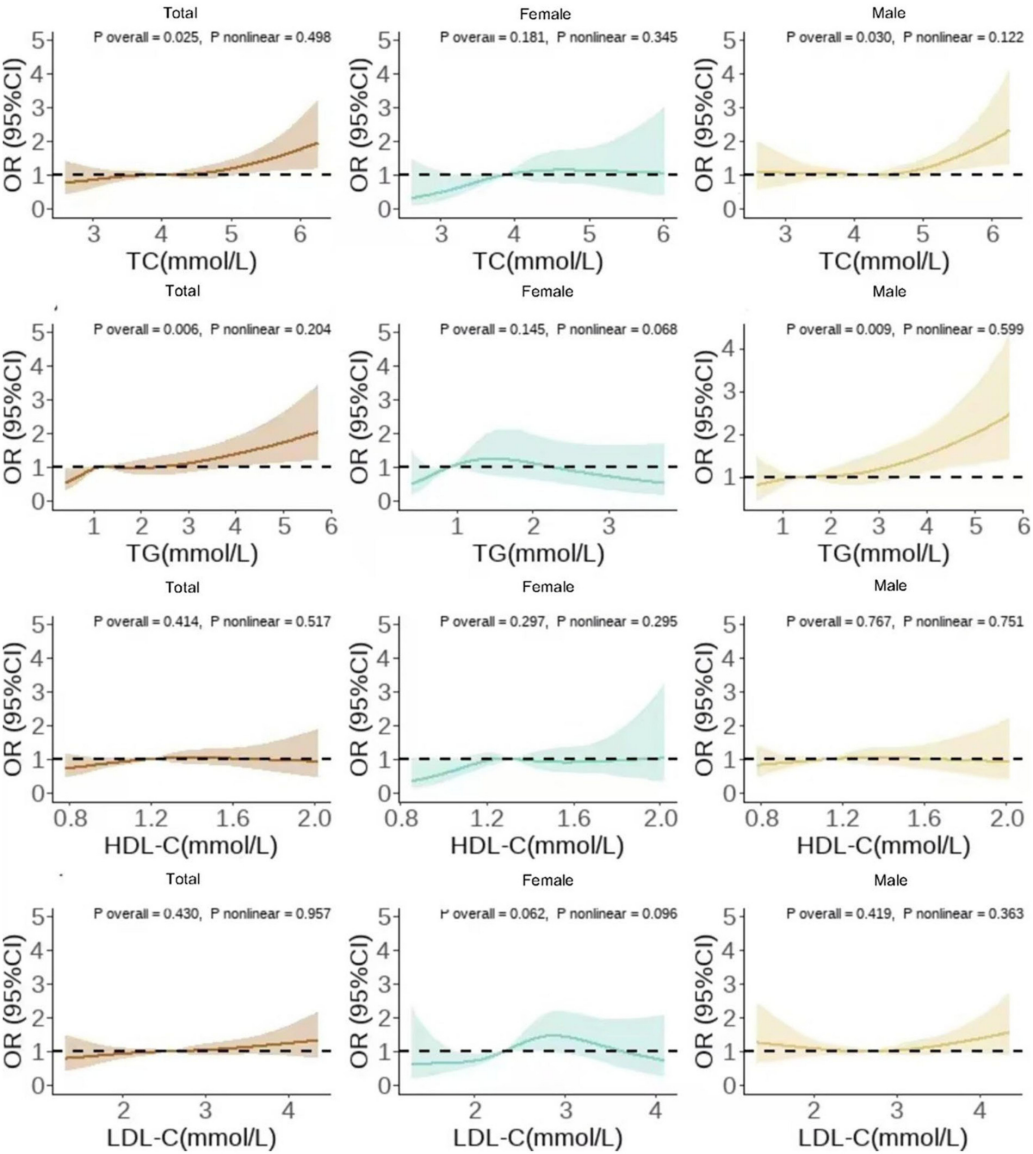
Dose-response relationship diagram between different lipid markers and hypertension onset.

Restricted cubic spline analysis, adjusted for all covariates, revealed that in the total population and among men, TC and TG levels showed a significant positive linear dose–response relationship with hypertension risk (*P _total_* < 0.05, *P _nonlinear_* > 0.05). No significant linear association was observed among women. HDL-C and LDL-C levels did not show a significant dose–response trend (*P* > 0.05) (Figure 1).

### 3.5 Lipid clusters and hypertension

Meanwhile, In the general population, the incidence rates of hypertension were 12.02%, 18.90%, 23.25%, and 33.58% when the number of lipid abnormalities clustered at 0, 1, 2, and ≥3, respectively, showing an increasing trend (*P _trend_* < 0.001). Among males, the incidence rates were 18.19%, 20.61%, 26.43%, and 36.44% for 0, 1, 2, and ≥3 clusters, respectively, showing an increasing trend (*P _trend_* < 0.001). No significant trend was observed among the female subjects (*P _trend_* = 0.059) (Supplementary Table S3). The results of Model 3 indicate that among men, the risk of hypertension onset was highest when the number of lipid abnormalities clustered ≥3 (OR = 1.719, 95% CI = 1.098–2.690, *P* = 0.018), with risk increasing as the number of clusters rose (*P _for trend_* = 0.044). In the overall population, risk increased only when the number of clusters was ≥3 (OR = 1.656, 95% CI = 1.089–2.518, *P* = 0.018), with no significant trend (*P _trend_* = 0.091). No significant association was observed in women (*P* > 0.05) (Supplementary Table S4).

### 3.6 Non-traditional lipid indicators and hypertension

**Figure 2:**
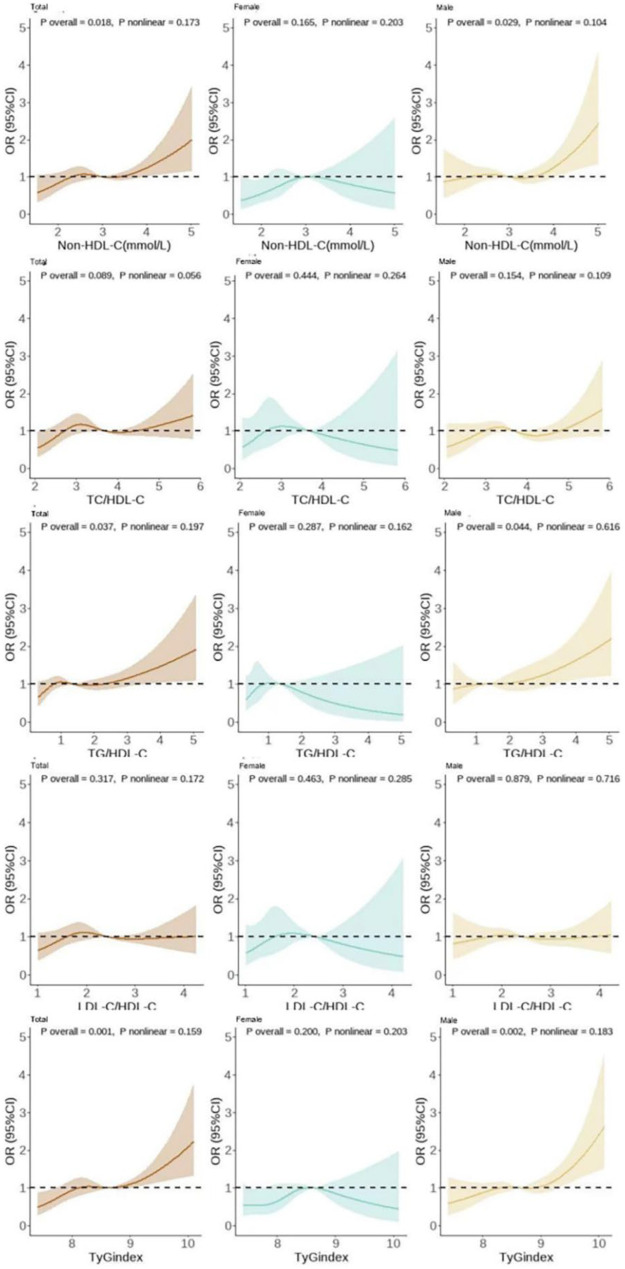
Dose-response relationship diagram between different non-traditional lipid markers and hypertension onset.

A thorough analysis of non-traditional lipid markers was conducted, revealing that in the general population, the incidence rates of hypertension at the Q4 level for non-HDL-C, TG/HDL-C, and TyG index were 23.65%, 25.23%, and 26.23%, respectively. These figures were all significantly higher than those at Q1 (9.08%, 7.27%, and 5.76%, *P _trend_* < 0.001). The results for males were consistent with the overall population, while for females, Q4 incidence rates for all indicators exceeded those of Q1 (*P _trend_* < 0.05) (Supplementary Table S5). The findings from Model 3 indicated that, within the general population, a one-unit rise in non-HDL-C, TG/HDL-C, and TyG index was associated with a 22.2% increase in the odds ratio (OR = 1.222, 95% CI = 1.050, 1.423), a 13.9% increase in the OR (OR = 1.139, 95% CI = 1.019–1.273), and a 46.5% increase in the OR (OR = 1.465, 95% CI = 1.194–1.796, *P* < 0.05), respectively. The results obtained for the male population were found to be consistent with the overall population (*P* < 0.05), while no significant associations were observed among the female population (*P* > 0.05) (Supplementary Table S6). RCS analysis revealed the following: In the total cohort and among males, non-HDL-C, TG/HDL-C, and TyG index levels exhibited a positive linear dose-response relationship with hypertension incidence risk (*P _total_* < 0.05, *P _nonlinear_* > 0.05); no significant linear relationship was observed in females (*P* > 0.05) (Figure 2).

## 4 Discussion

Our study examined the associations between blood lipid profiles, body mass index (BMI), and incident hypertension in a cohort of petrochemical industry employees. We found that abnormal LDL-C, elevated TC and TG levels, and clustering of dyslipidemia were associated with an increased risk of hypertension, particularly among male workers. In addition, non-traditional lipid markers, including non-HDL-C and the TyG index, showed consistent associations with hypertension risk. The coexistence of overweight or obesity with dyslipidemia was associated with a further increase in risk, highlighting the potential value of integrated metabolic risk profiling in occupational health surveillance settings.

Abnormal LDL-C has been identified as a key risk factor for hypertension in male employees: The study found a positive correlation between abnormal LDL-C levels and hypertension in the overall population and among males, which is consistent with the findings of a Japanese occupational cohort study (28% increased hypertension risk in men with elevated LDL-C) (22). A potential mechanism involves LDL-C upregulating angiotensin type 1 receptor (AT1) expression, leading to vasoconstriction and elevated blood pressure. The potential of statins to reduce both LDL-C and blood pressure by downregulating AT1 receptors lends further support to the hypothesis of a causal relationship (23, 24). This finding indicates that petrochemical enterprises should prioritise the monitoring of LDL-C levels in male employees, aiming to maintain them below 3.40 mmol/L.

The pronounced association between adverse lipid profiles and incident hypertension observed predominantly in male workers warrants specific consideration. Physiologically, this disparity may be heavily influenced by the vasoprotective and lipid-modulating effects of endogenous estrogen in premenopausal women, which enhances endothelial nitric oxide production and mitigates lipid-induced vascular damage (25, 26). In contrast, men lack this hormonal protection, making their vasculature more susceptible to the hypertensive effects of dyslipidemia. Furthermore, lifestyle and occupational factors likely compound this biological vulnerability. In the petrochemical industry, male employees often occupy roles with higher physical demands and occupational stress (such as oil extraction positions), and historically exhibit higher rates of adverse health behaviors, including smoking and alcohol consumption. These intersecting physiological and behavioral factors likely explain the heightened vulnerability of male workers to lipid-driven hypertension.

The linear association between TC and TG levels and hypertension warrants further consideration: In the general population, the highest risk of hypertension was observed at TC and TG levels in the Q4 quartile, and RCS analysis demonstrated a positive linear relationship between both lipids and hypertension. This phenomenon may be attributed to chronically elevated levels of TC and TG lead to vascular endothelial cell damage, which in turn impairs vascular diastolic function and ultimately results in hypertension (27, 28). It is recommended that TC and TG levels among employees be controlled below 4.55 mmol/L and 1.84 mmol/L, respectively (corresponding to the Q3 level).

The clustering of dyslipidaemia in men is demonstrated to significantly amplify the risk of hypertension: The highest risk is observed when men have ≥3 dyslipidemic risk factors, consistent with findings from the Iranian middle-aged and elderly cohort study (where dyslipidemia clustering doubled cardiovascular disease risk) (29). This finding indicates that male employees exhibiting multiple concurrent dyslipidemic abnormalities necessitate expeditious intervention to avert accumulating risk.

Non-traditional lipid markers serve as supplementary monitoring indicators: non-HDL-C (incorporating residual cholesterol) and TyG index (reflecting insulin resistance) have been demonstrated to have a strong association with hypertension (30, 31). This finding is consistent with the results of the Chinese Community Cohort Study (57% increased hypertension risk in elevated non-HDL-C individuals) (32). The absence of the necessity for additional testing renders these indicators well-suited for incorporation into health monitoring systems utilised by petrochemical workers. The calculation of these indicators from routine lipid and glucose data is a direct process, thus facilitating seamless integration into existing systems.

It is imperative that employees who are overweight or obese prioritise the prevention of dyslipidaemia: The combined effect of overweight/obesity and dyslipidaemia has been demonstrated to elevate hypertension risk. Despite the absence of observed additive interactions, it is recommended that overweight/obese employees (BMI ≥ 24.00 kg/m²) implement dietary control and exercise interventions to prevent dyslipidaemia and reduce hypertension risk (33–35).

When interpreting the multivariable models, it is important to distinguish between potential confounders and mediators. While Model 2 adjusted for standard demographic and baseline variables, Model 3 further adjusted for uric acid and liver function enzymes (alanine aminotransferase, alkaline phosphatase). We acknowledge that these additional variables are heavily intertwined with lipid metabolism and often cluster together in metabolic syndrome. Therefore, they may lie on the causal pathway between dyslipidemia and hypertension rather than acting merely as confounders. The attenuation of statistical significance for several lipid markers in Model 3 suggests a potential overadjustment. Consequently, we consider Model 2 to be the primary model for evaluating these associations, whereas Model 3 serves as an exploratory, highly conservative estimate.

Several limitations should be acknowledged. First, the retrospective design limited the availability of certain confounding variables, including detailed lifestyle factors and quantitative occupational exposure information, which may have resulted in residual confounding. Additionally, because the data was drawn from routine occupational health screenings, we were unable to systematically track and exclude incident cases of secondary hypertension or major cardiovascular and renal comorbidities that developed during the follow-up period, which could influence blood pressure trajectories. Furthermore, while we adjusted for continuous FPG to capture the full spectrum of glycemic dysregulation, and uric acid as an indicator of metabolic risk, the absence of calculated estimated glomerular filtration rate (eGFR) limits our ability to comprehensively account for baseline renal function. Second, the study was conducted at a single medical examination center in Xi'an, which may limit the generalizability of the findings. Third, the relatively short follow-up period and the lower proportion of female participants reduced statistical power for some subgroup analyses. Fourth, the reliance on periodic health examinations meant that the exact time of hypertension onset was unavailable, precluding the use of time-to-event survival analyses and limiting our ability to establish strong causal timelines.

## 5 Conclusion

In this occupational cohort, adverse lipid profiles, including elevated LDL-C, TC, and TG levels, as well as clustering of dyslipidemia, were associated with an increased risk of hypertension, particularly among male workers. Non-traditional lipid markers, such as non-HDL-C and the TyG index, showed consistent dose–response associations with hypertension. Overweight or obesity further amplified hypertension risk when coexisting with dyslipidemia. These findings suggest that comprehensive lipid profiling and consideration of combined metabolic risk factors may be useful for identifying individuals at higher risk of hypertension during routine occupational health screenings, even in the absence of quantitative environmental exposure data.

## Data Availability

The raw data supporting the conclusions of this article will be made available by the authors, without undue reservation.
