# Supplementary material for: Association between blood lipid levels, BMI, and hypertension among employees in petrochemical enterprises
Source: Front Cardiovasc Med. 2026 Apr 1;13:1788552. doi: 10.3389/fcvm.2026.1788552 (PMC13079123; doi:10.3389/fcvm.2026.1788552)
Supplement: Supplementary file 1 [file Table1.docx]

Supplementary material

**Fig S1** Participant flow diagram in this study.

**Fig S2** Dose-response relationship diagram between different non-traditional lipid markers and hypertension onset

**Table S1** Comparison of Baseline Characteristics Between the Normal Weight Group and the Overweight and Obese Group

**Table S2** Incidence of Hypertension in Different Dyslipidemic Conditions

**Table S3** The Impact of the Aggregation Degree of Dyslipidemia on Hypertension Incidence

**Table S4** The Impact of the Severity of Dyslipidemia on the Risk of Hypertension Onset

**Table S5** The Impact of Different Levels of Composite Indicators on Hypertension Incidence

**Table S6** Relationship Between Different Composite Indicators and Hypertension Risk in Model 3


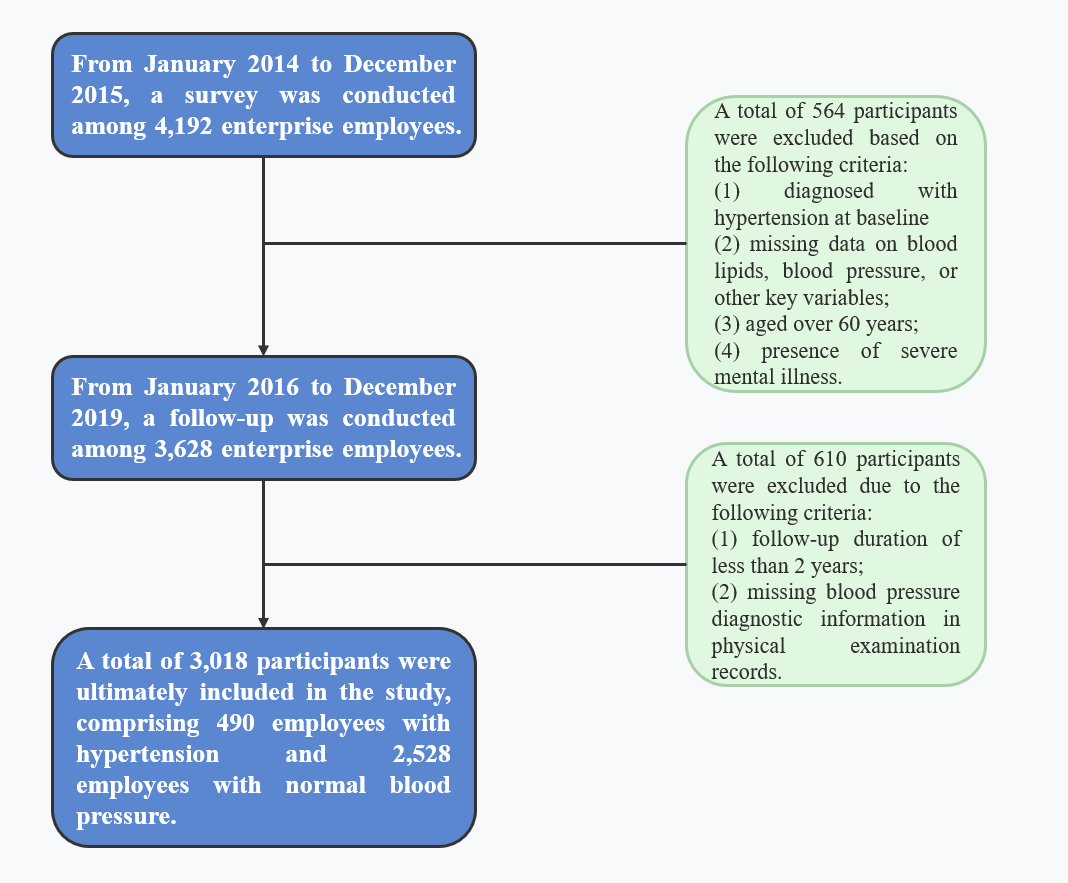


**Fig S1** Participant flow diagram in this study.


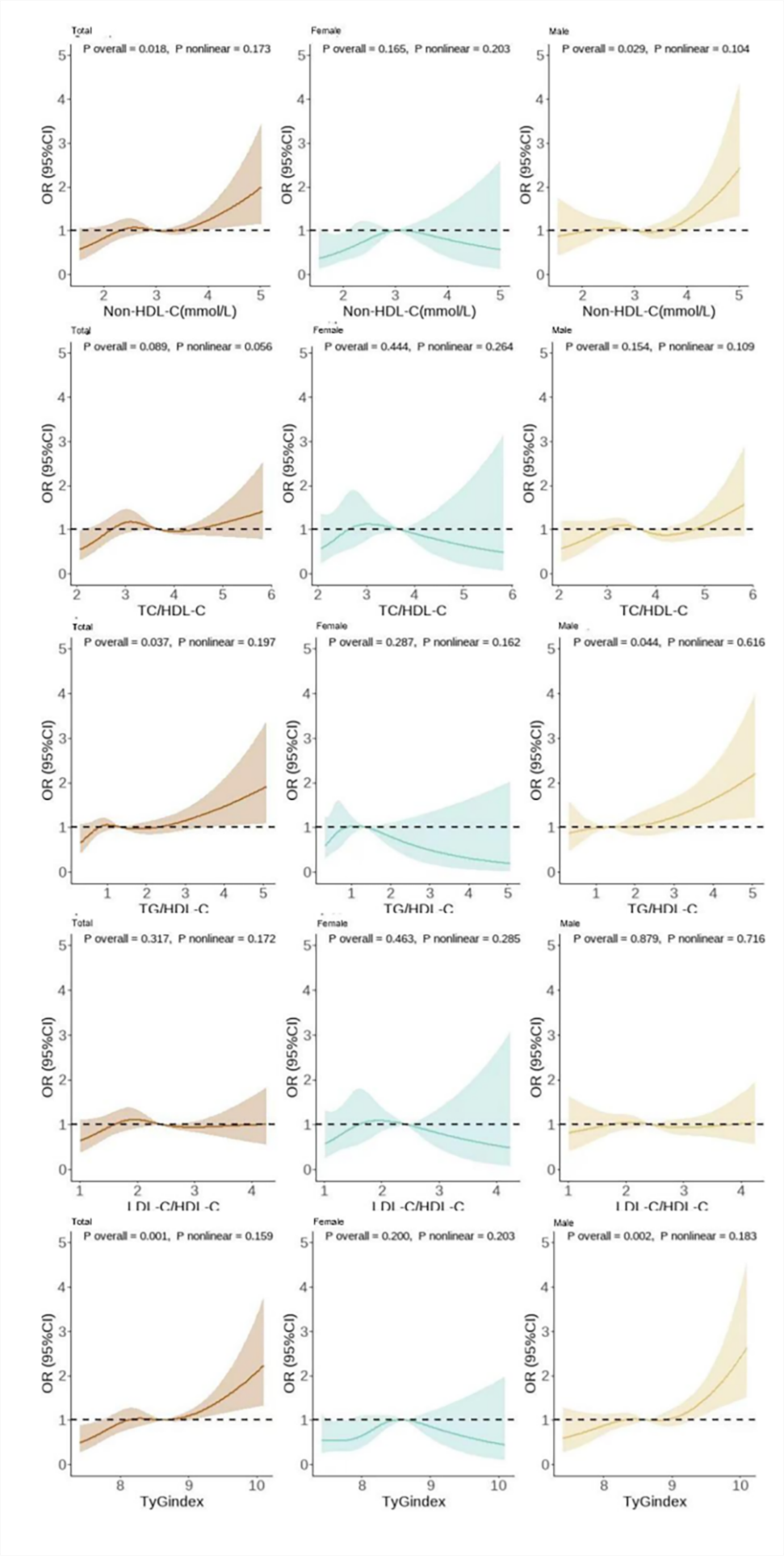


**Fig S2** Dose-response relationship diagram between different non-traditional lipid markers and hypertension onset

**Table S1** Comparison of Baseline Characteristics Between the Normal Weight Group and the Overweight and Obese Group

| **Characteristic** | BMI | | *P* |
| --- | --- | --- | --- |
|  | Normal | Abnormal |  |
| Gender |  |  | <0.001 |
| Female | 994 (52.84) | 255 (22.43) |  |
| Male | 887 (47.16) | 882 (77.57) |  |
| Marriage |  |  | <0.001 |
| Married | 1489 (79.16) | 990 (87.07) |  |
| Unmarried/Other | 392 (20.84) | 147 (12.93) |  |
| Job type |  |  | <0.001 |
| Oil production position | 1202 (63.90) | 835 (73.44) |  |
| Transportation/Station control position | 353 (18.77) | 119 (10.47) |  |
| Others | 326 (17.33) | 183 (16.09) |  |
| Diabetes |  |  | <0.001 |
| No | 1855 (98.62) | 1109 (97.54) |  |
| Yes | 26 (1.38) | 28 (2.46) |  |
| Carotid atherosclerosis |  |  | <0.001 |
| No | 1719 (91.39) | 968 (85.14) |  |
| Yes | 162 (8.61) | 169 (14.86) |  |
| Age (years) | 35.29±8.11 | 38.07±8.35 | <0.001 |
| Age Group (years) |  |  | <0.001 |
| 20-29 | 559 (29.72) | 204 (17.94) |  |
| 30-39 | 771 (40.99) | 466 (40.99) |  |
| ≥40 | 551 (29.29) | 467 (41.07) |  |
| Dyslipidemia |  |  | <0.001 |
| No | 1349 (71.72) | 365 (32.10) |  |
| Yes | 532 (28.28) | 772 (67.90) |  |
| SBP (mmHg) | 110.86±11.58 | 118.37±10.85 | <0.001 |
| DBP (mmHg) | 67.69±8.49 | 72.89±8.08 | <0.001 |
| FPG (mmol/L) | 4.57±0.52 | 4.73±0.64 | <0.001 |
| ALP (U/L) | 64.68±18.27 | 71.78±18.11 | <0.001 |
| GPT (U/L) | 16.85±11.07 | 26.54±16.18 | <0.001 |
| GOT (U/L) | 23.99±6.76 | 27.85±8.38 | <0.001 |
| GGT (U/L) | 22.22±18.75 | 38.20±28.39 | <0.001 |
| Urea (mmol/L) | 4.74±1.30 | 5.08±1.29 | <0.001 |
| Uric acid (umol/L) | 297.15±80.53 | 363.81±84.22 | <0.001 |
| Creatinine (umol/L) | 82.00±11.27 | 88.18±10.65 | <0.001 |

**Table S2** Incidence of Hypertension in Different Dyslipidemic Conditions

| Lipid Profile | General population | | | Female | | | Male | | |
| --- | --- | --- | --- | --- | --- | --- | --- | --- | --- |
|  |  |  |  |  |  |  |  |  |  |
|  | Total | Patient | Incidence rate (%) | Total | Patient | Incidence rate (%) | Total | Patient | Incidence rate (%) |
|  |  |  |  |  |  |  |  |  |  |
| TC |  |  |  |  |  |  |  |  |  |
| Normal | 2789 | 430 | 15.42 | 1193 | 99 | 8.3 | 1596 | 331 | 20.74 |
| Abnormal | 229 | 60 | 26.2 | 56 | 6 | 10.71 | 173 | 54 | 31.21 |
| *χ2* |  | 18.093 |  |  | 0.152 |  |  | 10.057 |  |
| *P* |  | <0.001^a^ |  |  | 0.696 |  |  | 0.002^a^ |  |
| TG |  |  |  |  |  |  |  |  |  |
| Normal | 2143 | 275 | 12.83 | 1113 | 86 | 7.73 | 1030 | 189 | 18.35 |
| Abnormal | 875 | 215 | 24.57 | 136 | 19 | 13.97 | 739 | 196 | 26.52 |
| *χ2* |  | 62.956 |  |  | 6.136 |  |  | 16.879 |  |
| *P* |  | <0.001^a^ |  |  | 0.013^a^ |  |  | <0.001^a^ |  |
| HDL-C |  |  |  |  |  |  |  |  |  |
| Normal | 2480 | 380 | 15.32 | 1147 | 96 | 8.37 | 1333 | 284 | 21.31 |
| Abnormal | 538 | 110 | 20.45 | 102 | 9 | 8.82 | 436 | 101 | 23.17 |
| *χ2* |  | 8.533 |  |  | 0.025 |  |  | 0.667 |  |
| *P* |  | 0.003^a^ |  |  | 0.874 |  |  | 0.414 |  |
| LDL-C |  |  |  |  |  |  |  |  |  |
| Normal | 2672 | 400 | 14.97 | 1171 | 95 | 8.11 | 1501 | 305 | 20.32 |
| Abnormal | 346 | 90 | 26.01 | 78 | 10 | 12.82 | 268 | 80 | 29.85 |
| *χ2* |  | 27.461 |  |  | 2.105 |  |  | 12.132 |  |
| *P* |  | <0.001^a^ |  |  | 0.147 |  |  | <0.001^a^ |  |

Note: ^a^ Statistically significant at 0.05 level

**Table S3** The Impact of the Aggregation Degree of Dyslipidemia on Hypertension Incidence

| Clustering of Dyslipidemia | General population | | | Female | | | Male | | |
| --- | --- | --- | --- | --- | --- | --- | --- | --- | --- |
|  | Total | Patient | Incidence rate (%) | Total | Patient | Incidence rate (%) | Total | Patient | Incidence rate (%) |
| 0 | 1714 | 206 | 12.02 | 983 | 73 | 7.43 | 731 | 133 | 18.19 |
| 1 | 767 | 145 | 18.90 | 180 | 24 | 13.33 | 587 | 121 | 20.61 |
| 2 | 400 | 93 | 23.25 | 67 | 5 | 7.46 | 333 | 88 | 26.43 |
| ≥3 | 137 | 46 | 33.58 | 19 | 3 | 15.79 | 118 | 43 | 36.44 |
| *χ2* |  | 69.755 |  |  | 3.558 |  |  | 22.439 |  |
| *P* _trend_ |  | <0.001^a^ |  |  | 0.059 |  |  | <0.001^a^ |  |

Note: ^a^ Statistically significant at 0.05 level

**Table S4** The Impact of the Severity of Dyslipidemia on the Risk of Hypertension Onset

| Clustering of Dyslipidemia | Model 1 | | Model 2 | | Model 3 | |
| --- | --- | --- | --- | --- | --- | --- |
|  | OR (95%CI) | *P* | OR (95%CI) | *P* | OR (95%CI) | P |
| Total |  |  |  |  |  |  |
| 0 | — |  | — |  | — |  |
| 1 | 1.707 (1.353, 2.152) | <0.001 | 0.964 (0.746, 1.244) | 0.776 | 0.944 (0.729, 1.222) | 0.661 |
| 2 | 2.218 (1.686, 2.916) | <0.001 | 1.083 (0.800, 1.468) | 0.605 | 1.045 (0.770, 1.419) | 0.776 |
| ≥3 | 3.700 (2.522, 5.429) | <0.001 | 1.751 (1.162, 2.640) | 0.007 | 1.656 (1.089, 2.518) | 0.018 |
| *P _trend_* | <0.001^a^ |  | 0.042^a^ |  | 0.091 |  |
| Female |  |  |  |  |  |  |
| 0 | — |  | — |  | — |  |
| 1 | 1.918 (1.173, 3.135) | 0.009 | 1.188 (0.699, 2.021) | 0.524 | 1.072 (0.620, 1.853) | 0.803 |
| 2 | 1.005 (0.392, 2.578) | 0.991 | 0.723 (0.274, 1.903) | 0.511 | 0.606 (0.226, 1.625) | 0.320 |
| ≥3 | 2.337 (0.666, 8.207) | 0.185 | 1.314 (0.354, 4.884) | 0.683 | 1.175 (0.308, 4.487) | 0.814 |
| *P _trend_* | 0.061 |  | 0.938 |  | 0.697 |  |
| Male |  |  |  |  |  |  |
| 0 | — |  | — |  | — |  |
| 1 | 1.167 (0.887, 1.536) | 0.268 | 0.917 (0.687, 1.223) | 0.554 | 0.908 (0.678, 1.215) | 0.515 |
| 2 | 1.615 (1.187, 2.197) | 0.002 | 1.138 (0.820, 1.579) | 0.440 | 1.114 (0.801, 1.550) | 0.521 |
| ≥3 | 2.578 (1.695, 3.921) | <0.001 | 1.803 (1.165, 2.792) | 0.008 | 1.719 (1.098, 2.690) | 0.018 |
| *P _trend_* | <0.001^a^ |  | 0.024^a^ |  | 0.044^a^ |  |

Notes: — : reference group

^a^ Statistically significant at 0.05 level

Model 1: No variables adjusted; Model 2: Adjusted for age, gender, marital status, BMI, occupation, carotid atherosclerosis, and FPG; Model 3: Adjusted for age, gender, marital status, BMI, occupation, carotid atherosclerosis, FPG, uric acid, alanine aminotransferase, and alkaline phosphatase. When stratified by gender, gender was not adjusted in Models 2 and 3.

**Table S5** The Impact of Different Levels of Composite Indicators on Hypertension Incidence

| Indicator | General population | | | Female | | | Male | | |
| --- | --- | --- | --- | --- | --- | --- | --- | --- | --- |
|  | Total | Patient | Incidence rate (%) | Total | Patient | Incidence rate (%) | Total | Patient | Incidence rate (%) |
| Non-HDL-C |  |  |  |  |  |  |  |  |  |
| Q1 (1.13, 2.32) | 738 | 67 | 9.08 | 466 | 22 | 4.72 | 272 | 45 | 16.54 |
| Q2 (2.33, 2.79) | 770 | 115 | 14.94 | 367 | 35 | 9.54 | 403 | 80 | 19.85 |
| Q3 (2.80, 3.31) | 753 | 129 | 17.13 | 253 | 24 | 9.49 | 500 | 105 | 21.00 |
| Q4 (3.32, 5.32) | 757 | 179 | 23.65 | 163 | 24 | 14.72 | 594 | 155 | 26.09 |
| *χ2* |  | 57.91 |  |  | 15.642 |  |  | 11.137 |  |
| *P _trend_* |  | <0.001 |  |  | <0.001 |  |  | 0.001 |  |
| TC/HDL-C |  |  |  |  |  |  |  |  |  |
| Q1 (1.56, 2.79) | 752 | 58 | 7.71 | 526 | 28 | 5.32 | 226 | 30 | 13.27 |
| Q2 (2.80, 3.34) | 760 | 110 | 14.47 | 391 | 40 | 10.23 | 369 | 70 | 18.97 |
| Q3 (3.35, 3.97) | 748 | 149 | 19.92 | 221 | 25 | 11.31 | 527 | 124 | 23.53 |
| Q4 (3.98, 6.96) | 758 | 173 | 22.82 | 111 | 12 | 10.81 | 647 | 161 | 24.88 |
| *χ2* |  | 71.426 |  |  | 8.568 |  |  | 14.63 |  |
| *P _trend_* |  | <0.001 |  |  | 0.003 |  |  | <0.001 |  |
| TG/HDL-C |  |  |  |  |  |  |  |  |  |
| Q1 (0.20, 0.61) | 743 | 54 | 7.27 | 543 | 29 | 5.34 | 200 | 25 | 12.50 |
| Q2 (0.62, 0.99) | 758 | 111 | 14.64 | 381 | 41 | 10.76 | 377 | 70 | 18.57 |
| Q3 (1.00, 1.67) | 760 | 134 | 17.63 | 227 | 22 | 9.69 | 533 | 112 | 21.01 |
| Q4 (1.68, 6.04) | 757 | 191 | 25.23 | 98 | 13 | 13.27 | 659 | 178 | 27.01 |
| *χ2* |  | 89.185 |  |  | 9.544 |  |  | 22.483 |  |
| *P _trend_* |  | <0.001 |  |  | 0.002 |  |  | <0.001 |  |
| LDL-C/HDL-C |  |  |  |  |  |  |  |  |  |
| Q1 (0.64, 1.67) | 754 | 63 | 8.36 | 535 | 29 | 5.42 | 219 | 34 | 15.53 |
| Q2 (1.67, 2.13) | 753 | 117 | 15.54 | 362 | 36 | 9.94 | 391 | 81 | 20.72 |
| Q3 (2.14, 2.67) | 755 | 142 | 18.81 | 227 | 26 | 11.45 | 528 | 116 | 21.97 |
| Q4 (2.68, 5.60) | 756 | 168 | 22.22 | 125 | 14 | 11.20 | 631 | 154 | 24.41 |
| *χ2* |  | 55.852 |  |  | 9.198 |  |  | 7.237 |  |
| *P _trend_* |  | <0.001 |  |  | 0.002 |  |  | 0.007 |  |
| TyG index |  |  |  |  |  |  |  |  |  |
| Q1 (7.09, 7.99) | 747 | 43 | 5.76 | 517 | 24 | 4.64 | 230 | 19 | 8.26 |
| Q2 (7.98, 8.37) | 760 | 112 | 14.74 | 402 | 38 | 9.45 | 358 | 74 | 20.67 |
| Q3 (8.38, 8.82) | 760 | 138 | 18.16 | 220 | 25 | 11.36 | 540 | 113 | 20.93 |
| Q4 (8.83, 10.45) | 751 | 197 | 26.23 | 110 | 18 | 16.36 | 641 | 179 | 27.93 |
| *χ2* |  | 115.755 |  |  | 20.921 |  |  | 34.375 |  |
| *P _trend_* |  | <0.001 |  |  | <0.001 |  |  | <0.001 |  |

Notes: Non-HDL-C: TC minus HDL-C

TC/HDL-C: Refers to the ratio of TC to HDL-C

TG/HDL-C: Refers to the ratio of TG to HDL-C

LDL-C/HDL-C: Refers to the ratio of LDL-C to HDL-C

TyG index: Take the logarithm of the product of TG (mg/dL) and FPG (mg/dL) to obtain the TyG index value.

**Table S6** Relationship Between Different Composite Indicators and Hypertension Risk in Model 3

| Indicator | General population | | Female | | Male | |
| --- | --- | --- | --- | --- | --- | --- |
|  | OR (95% CI) | *P* | OR (95% CI) | *P* | OR (95% CI) | *P* |
| Non-HDL-C | 1.222 (1.050, 1.423) | 0.010 | 1.270 (0.924, 1.745) | 0.141 | 1.199 (1.008, 1.425) | 0.040 |
| Q1 | — |  | — |  | — |  |
| Q2 | 1.389 (0.997, 1.934) | 0.052 | 1.809 (1.031, 3.176) | 0.039 | 1.164 (0.770, 1.760) | 0.472 |
| Q3 | 1.156 (0.829, 1.613) | 0.392 | 1.396 (0.752, 2.590) | 0.290 | 0.998 (0.669, 1.490) | 0.994 |
| Q4 | 1.429 (1.028, 1.986) | 0.034 | 1.978 (1.041, 3.757) | 0.037 | 1.204 (0.815, 1.777) | 0.351 |
| *P trend* | 0.091 | | 0.073 | | 0.406 | |
| TC/HDL-C | 1.076 (0.928, 1.248) | 0.333 | 1.031 (0.739, 1.438) | 0.858 | 1.080 (0.916, 1.274) | 0.359 |
| Q1 | — |  | — |  | — |  |
| Q2 | 1.418 (—, 2.012) | 0.050 | 1.486 (0.883, 2.503) | 0.136 | 1.360 (0.844, 2.193) | 0.207 |
| Q3 | 1.424 (1.004, 2.018) | 0.047 | 1.177 (0.642, 2.158) | 0.599 | 1.491 (0.950, 2.340) | 0.082 |
| Q4 | 1.204 (0.837, 1.734) | 0.317 | 0.934 (0.430, 2.027) | 0.862 | 1.250 (0.794, 1.969) | 0.335 |
| *P trend* | 0.773 | | 0.917 | | 0.781 | |
| TG/HDL-C | 1.139 (1.019, 1.273) | 0.022 | 0.916 (0.653, 1.284) | 0.609 | 1.174 (1.042, 1.322) | 0.008 |
| Q1 | — |  | — |  | — |  |
| Q2 | 1.516 (1.062, 2.163) | 0.022 | 1.649 (0.985, 2.763) | 0.057 | 1.327 (0.805, 2.187) | 0.267 |
| Q3 | 1.236 (0.854, 1.790) | 0.261 | 1.056 (0.564, 1.976) | 0.865 | 1.202 (0.739, 1.955) | 0.458 |
| Q4 | 1.473 (1.007, 2.154) | 0.046 | 1.235 (0.566, 2.696) | 0.596 | 1.431 (0.881, 2.323) | 0.147 |
| *P trend* | 0.213 | | 0.949 | | 0.188 | |
| LDL-C/HDL-C | 1.012 (0.857, 1.196) | 0.886 | 1.061 (0.734, 1.533) | 0.754 | 0.992 (0.824, 1.195) | 0.934 |
| Q1 | — |  | — |  | — |  |
| Q2 | 1.383 (0.983, 1.944) | 0.062 | 1.546 (0.915, 2.614) | 0.104 | 1.257 (0.798, 1.979) | 0.324 |
| Q3 | 1.253 (0.888, 1.767) | 0.199 | 1.392 (0.773, 2.509) | 0.271 | 1.148 (0.742, 1.778) | 0.535 |
| Q4 | 1.137 (0.798, 1.620) | 0.479 | 0.986 (0.473, 2.054) | 0.969 | 1.080 (0.698, 1.669) | 0.731 |
| *P trend* | 0.977 | | 0.828 | | 0.780 | |
| TyG index | 1.465 (1.194, 1.196) | <0.001 | 1.296 (0.845, 1.989) | 0.235 | 1.500 (1.188, 1.893) | 0.001 |
| Q1 | — |  | — |  | — |  |
| Q2 | 2.039 (1.400, 2.971) | <0.001 | 1.804 (1.049, 3.102) | 0.033 | 2.244 (1.310, 3.845) | 0.003 |
| Q3 | 1.798 (1.222, 2.645) | 0.003 | 1.622 (0.867, 3.036) | 0.130 | 1.883 (1.114, 3.182) | 0.018 |
| Q4 | 2.280 (1.537, 3.383) | <0.001 | 2.178 (1.063, 4.465) | 0.033 | 2.399 (1.419, 4.057) | 0.001 |
| *P trend* | <0.001 | | 0.040 | | 0.011 | |
